# CRISPR-Cas: biology, mechanisms and relevance

**DOI:** 10.1098/rstb.2015.0496

**Published:** 2016-11-05

**Authors:** Frank Hille, Emmanuelle Charpentier

**Affiliations:** 1 Department of Regulation in Infection Biology, Max Planck Institute for Infection Biology, Berlin 10117, Germany; 2 The Laboratory for Molecular Infection Medicine Sweden (MIMS), Umeå Centre for Microbial Research (UCMR), Department of Molecular Biology, Umeå University, Umeå 90187, Sweden

**Keywords:** CRISPR, Cas9, bacteriophage, genome editing

## Abstract

Prokaryotes have evolved several defence mechanisms to protect themselves from viral predators. Clustered regularly interspaced short palindromic repeats (CRISPR) and their associated proteins (Cas) display a prokaryotic adaptive immune system that memorizes previous infections by integrating short sequences of invading genomes—termed spacers—into the CRISPR locus. The spacers interspaced with repeats are expressed as small guide CRISPR RNAs (crRNAs) that are employed by Cas proteins to target invaders sequence-specifically upon a reoccurring infection. The ability of the minimal CRISPR-Cas9 system to target DNA sequences using programmable RNAs has opened new avenues in genome editing in a broad range of cells and organisms with high potential in therapeutical applications. While numerous scientific studies have shed light on the biochemical processes behind CRISPR-Cas systems, several aspects of the immunity steps, however, still lack sufficient understanding. This review summarizes major discoveries in the CRISPR-Cas field, discusses the role of CRISPR-Cas in prokaryotic immunity and other physiological properties, and describes applications of the system as a DNA editing technology and antimicrobial agent.

This article is part of the themed issue ‘The new bacteriology’.

## 1. Introduction

Being the most abundant entities on our planet, bacterial and archaeal viruses (bacteriophages or phages) display a constant threat to prokaryotic life. In order to withstand phages, prokaryotes have evolved several defence strategies. In the past decade, the prokaryotic immune system CRISPR-Cas (clustered regularly interspaced short palindromic repeats-CRISPR-associated) caught increasing attention in the scientific community not only because of its unique adaptive nature, but also because of its therapeutic potential. This review seeks to summarize the major discoveries made in the field of CRISPR-Cas, and describes the biological roles of the system in antiviral defence and other biological pathways as well as its significance for medical application.

CRISPR-Cas is the only adaptive immune system in prokaryotes known so far. In this system, small guide RNAs (crRNAs) are employed for sequence-specific interference with invading nucleic acids. CRISPR-Cas comprises a genomic locus called CRISPR that harbours short repetitive elements (repeats) separated by unique sequences (spacers), which can originate from mobile genetic elements (MGEs) such as bacteriophages, transposons or plasmids. The so-called CRISPR array is preceded by an AT-rich leader sequence and is usually flanked by a set of *cas* genes encoding the Cas proteins [1–4]. To date, CRISPR-Cas systems can be divided into two main classes, which are further classified into six types and several sub-types [5–7]. The classification is based on the occurrence of effector Cas proteins that convey immunity by cleaving foreign nucleic acids. In class 1 CRISPR-Cas systems (types I, III and IV), the effector module consists of a multi-protein complex whereas class 2 systems (types II, V and VI) use only one effector protein [5].

## 2. Molecular mechanisms: adaptation, maturation and interference

**Figure 1.**
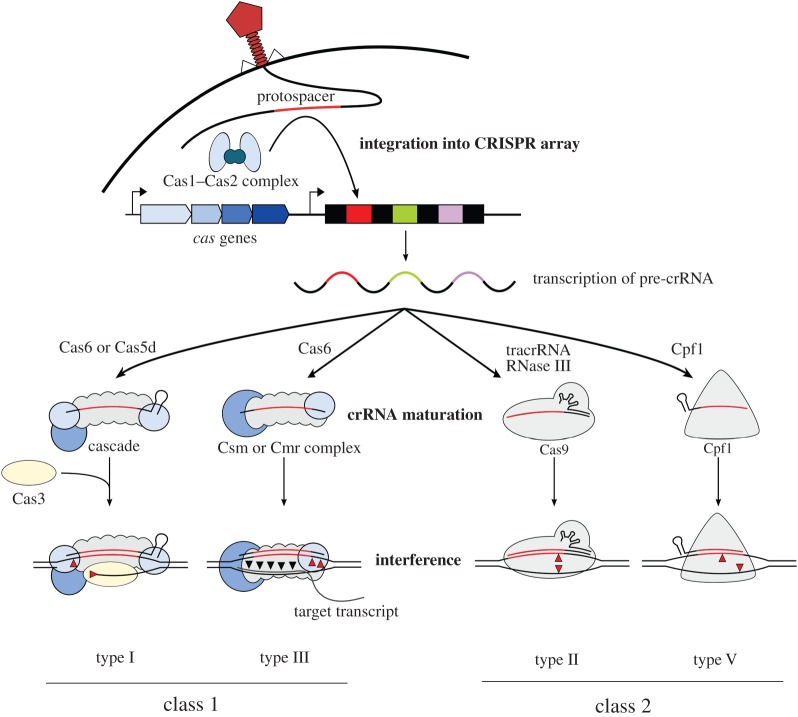
Simplified model of the immunity mechanisms of class 1 and class 2 CRISPR-Cas systems. The CRISPR-Cas systems are composed of a *cas* operon (blue arrows) and a CRISPR array that comprises identical repeat sequences (black rectangles) that are interspersed by phage-derived spacers (coloured rectangles). Upon phage infection, a sequence of the invading DNA (protospacer) is incorporated into the CRISPR array by the Cas1–Cas2 complex. The CRISPR array is then transcribed into a long precursor CRISPR RNA (pre-crRNA), which is further processed by Cas6 in type I and III systems (processing in type I-C CRISPR-Cas systems by Cas5d). In type II CRISPR-Cas systems, crRNA maturation requires tracrRNA, RNase III and Cas9, whereas in type V-A systems Cpf1 alone is sufficient for crRNA maturation. In the interference state of type I systems, Cascade is guided by crRNA to bind the foreign DNA in a sequence-specific manner and subsequently recruits Cas3 that degrades the displaced strand through its 3′–5′ exonucleolytic activity. Type III-A and type III-B CRISPR-Cas systems employ Csm and Cmr complexes, respectively, for cleavage of DNA (red triangles) and its transcripts (black triangles). A ribonucleoprotein complex consisting of Cas9 and a tracrRNA : crRNA duplex targets and cleaves invading DNA in type II CRISPR-Cas systems. The crRNA-guided effector protein Cpf1 is responsible for target degradation in type V systems. Red triangles represent the cleavage sites of the interference machinery.

The CRISPR-Cas system acts in a sequence-specific manner by recognizing and cleaving foreign DNA or RNA. The defence mechanism can be divided into three stages: (i) adaptation or spacer acquisition, (ii) crRNA biogenesis, and (iii) target interference (figure 1).

### (a) Adaptation

In a first phase, a distinct sequence of the invading MGE called a protospacer is incorporated into the CRISPR array yielding a new spacer. This event enables the host organism to memorize the intruder's genetic material and displays the adaptive nature of this immune system [1]. Two proteins, Cas1 and Cas2, seem to be ubiquitously involved in the spacer acquisition process as they can be found in almost all CRISPR-Cas types. Exceptions are the type III-C, III-D and IV CRISPR-Cas systems, which harbour no homologous proteins. Moreover, type V-C shows a minimal composition as it comprises only a putative effector protein termed C2C3 and a Cas1 homologue [5–7]. In past years, major advances have been made in revealing the biochemical and genetic principles of CRISPR-Cas immunity. However, the mechanism of spacer acquisition is still not fully understood [8,9]. The selection of protospacers and their processing before integration remain widely obscure in many CRISPR-Cas types. Recent findings, however, shed light on the biochemistry of the spacer integration process. It has been demonstrated that Cas1 and Cas2 of the type I-E system of *Escherichia coli* form a complex that promotes the integration of new spacers in a manner that is reminiscent of viral integrases and transposases [10–13]. Although both Cas1 and Cas2 are nucleases [14–16], the catalytically active site of Cas2 is dispensable for spacer acquisition [10–12]. A new spacer is usually incorporated at the leader-repeat boundary of the CRISPR array [1] while the first repeat of the array is duplicated [17,18].

The mechanisms of the different CRISPR-Cas types might be conserved only to a certain extent as several studies have shown variations regarding the requirements and targets of the adaptation machinery. While Cas1 and Cas2 are sufficient to promote spacer acquisition in most studied type I CRISPR-Cas systems, type I-B further requires Cas4 for adaptation [19]. The type I-F CRISPR-Cas system of *Pseudomonas aeruginosa* additionally requires the interference machinery to promote the uptake of new spacers [20]. Similarly, type II-A systems require Csn2, Cas9 and tracrRNA (*trans* activating CRISPR RNA—see further details below) for acquisition [1,21,22]. Another, so far unique, adaptation mode was revealed for a type III-B Cas1 protein that is fused to a reverse transcriptase. Here, acquisition from both DNA and RNA was reported [23].

The selection of a target sequence that is integrated into the CRISPR locus is not random. It has been demonstrated that in type I, II and V CRISPR-Cas systems, a short sequence, called the protospacer adjacent motif (PAM), is located directly next to the protospacer and is crucial for acquisition and interference [24–29]. In type II-A CRISPR-Cas systems, the PAM-recognizing domain of Cas9 is responsible for protospacer selection [21,22]. It is believed that after protospacer selection, Cas9 recruits Cas1, Cas2 and possibly Csn2 for integration of the new spacer into the CRISPR array. This feature may be conserved among all class 2 CRISPR-Cas systems although experimental evidence is missing. For type I-E, the Cas1–Cas2 complex is sufficient for spacer selection and integration although it has been reported that the presence of the interference complex increases the frequency of integrated spacers that are adjacent to a proper PAM [24,25]. Moreover, in a process called priming, the interference machinery of several type I CRISPR-Cas systems can stimulate the increased uptake of new spacers upon crRNA-guided binding to a protospacer that was selected upon a first infection [19,25,30]. This process displays a distinct adaptation mode compared to naive spacer acquisition as it strictly requires a pre-existing spacer matching the target. It usually leads to higher acquisition rates from protospacers that lie in close proximity to the target site [25]. Interestingly, primed spacer acquisition does not depend on target cleavage as it also functions for degenerated target sites that would usually result in impaired interference [31]. The exact mechanism remains obscure but it has been demonstrated that the interference complex can recruit Cas1 and Cas2 during PAM-independent binding to DNA [32].

### (b) Biogenesis

To enable immunity, the CRISPR array is transcribed into a long precursor crRNA (pre-crRNA) that is further processed into mature guide crRNAs containing the memorized sequences of invaders [33,34]. In type I and III systems, members of the Cas6 family perform the processing step yielding intermediate species of crRNAs that are flanked by a short 5′ tag. One exception is given by the type I-C systems, which do not code for Cas6 proteins. Here, the protein Cas5d processes pre-crRNA resulting in intermediate crRNAs with an 11 nt 5′ tag [33,35–38]. Further trimming of the 3′ end of the intermediate crRNA by an unknown nuclease can occur and yields mature crRNA species composed of a full spacer portion (5′ end) and a repeat-portion (3′ end), which usually displays a hairpin structure in most type I systems [39–41]. The maturation of crRNAs in class 2 CRISPR-Cas systems differs significantly. In type II systems, tracrRNA is required for the processing of the pre-crRNA. The anti-repeat sequence of this RNA enables the formation of an RNA duplex with each of the repeats of the pre-crRNA, which is stabilized by Cas9. The duplex is then recognized and processed by the host RNase III yielding an intermediate form of crRNA that undergoes further maturation by a still unknown mechanism to lead to the mature small guide RNA [42]. An RNase III-independent mechanism was discovered in the type II-C CRISPR-Cas system of *Neisseria meningitidis*. Here, promoter sequences were identified to lie within each repeat and some were able to initiate transcription leading to intermediate crRNA species. Even though RNase III-mediated 3′ processing of the crRNA : tracrRNA duplex was observed, it was dispensable for interference [43]. In the type V-A CRISPR-Cas system, it has been shown that Cpf1 has a dual function during CRISPR-Cas immunity. Cpf1 processes premature crRNAs [28] and, following a further maturation event of unknown nature, uses the processed crRNAs that it has generated to cleave target DNA [28,29].

### (c) Interference

In the last stage of immunity, mature crRNAs are used as guides to specifically interfere with the invading nucleic acids. Class 1 systems employ Cascade (CRISPR-associated complex for antiviral defence)-like complexes to achieve target degradation, while in class 2 systems, a single effector protein is sufficient for target interference [29,39,44–49]. To avoid self-targeting, type I, II and V systems specifically recognize the PAM sequence that is located upstream (types I and V) or downstream (type II) of the protospacer [26,28,29,31,45,50–52]. In type III systems, the discrimination between self and non-self is achieved via the 5′ tag of the mature crRNA, which must not base pair with the target to enable degradation by the complex [53].

In type I systems, Cascade localizes invading DNA in a crRNA-dependent manner and further recruits the nuclease Cas3 for target degradation. Cas3 induces a nick on the foreign DNA and subsequently degrades the target DNA [54,55]. In type II CRISPR-Cas systems, the tracrRNA:crRNA duplex guides the effector protein Cas9 to introduce a double-strand break in the target DNA [45]. The interference machinery of type III systems comprises Cas10-Csm (types III-A and III-D) and Cas10-Cmr (types III-B and III-C) complexes [5], which are able to target both DNA and RNA [38,39,47,49,56–63]. Intriguingly, it has been shown that interference of type III-A and type III-B systems depends on the transcription of the target DNA [57,58]. More precisely, the subunit Cas10 cleaves the DNA while Csm3 [59,60] and Cmr4 [61] cleave the transcribed mRNA in type III-A and type III-B CRISPR-Cas systems, respectively. Interference in type V CRISPR-Cas systems shows similarities to interference in type II. An RNA duplex, consisting of tracrRNA and crRNA, is strictly required for target cleavage in type V-B systems [7]. Type V-A, however, only employ crRNA for target localization and degradation [28,29].

## 3. Anti-CRISPR mechanisms

Prokaryotes harbour a remarkable arsenal of defence strategies in order to coexist with their viral predators (box 1). As a part of the constant arms race between bacteria and their viral counterparts, phages have evolved different strategies to overcome antiviral defence mechanisms. This paragraph summarizes the research on how phages evade the CRISPR-Cas systems.

Box 1. **CRISPR-Cas – What else? (Alternative defence mechanisms in bold type)**

Apart from CRISPR-Cas systems, prokaryotes have evolved a comprehensive set of defence mechanisms to protect themselves against predators. The viral infection cycle is initiated by adsorption of the phage onto the bacterial cell surface, where the phages recognize host-specific receptors on the outer membranes or cell walls of the host. Bacteria can **prevent phage adsorption** by producing an extracellular matrix that physically blocks the access to the specific receptor. Further counter-strategies involve mutating phage receptors and production of competitive inhibitors that occupy the receptor and thus lead to a reduced susceptibility to phage adsorption [64–66]. In the next step of infection, phages inject their genetic material into the host. In order to block the entry of viral DNA, bacteria use the so-called **superinfection exclusion (Sie)** systems that are often encoded by prophages. These systems comprise a set of proteins that prevent translocation of phage DNA into the cytoplasm [67,68].

Once entered, viral DNA can be degraded by **restriction-modification (RM)** systems that use nucleases to recognize and cleave short motifs present on the invading DNA. Non-methylated DNA is recognized by these restriction enzymes and self-cleavage is prevented by methylation of target sites on the host genome [69,70]. Another defence strategy blocks phage propagation by sacrificing an infected host cell, thus protecting the bacterial population. These **abortive infection (Abi)** mechanisms use proteins that sense infections and consequently induce cell death through, e.g. membrane depolarization, inhibiting the host's translational apparatus or exploiting components of toxin-antitoxin systems [71–74]. Less well-characterized antiviral systems encompass **bacteriophage exclusion (BREX)** and prokaryotic **Argonautes**. While BREX inhibits viral replication and DNA integration of lysogenic phages [75], Argonaute proteins are DNA- or RNA-guided nucleases that cleave invading DNA in a sequence-specific manner [76–78].

Phages can escape the CRISPR-Cas interference machinery through random mutations in the protospacer region or the PAM sequence [26,51]. As a counter strategy, several type I CRISPR-Cas systems show an elevated uptake of new spacers as a direct result of mismatches in the PAM or in the targeted protospacer during primed acquisition (see §2). Moreover, the efficiency of escaping CRISPR-Cas immunity by point mutations is strongly impaired in bacterial populations that show high spacer diversity. A possible explanation for this observation is that spacer diversity increases the adaptive pressure on the virus and thus leads to rapid extinction of the predator [79].

Recent studies demonstrated that Mu-like phages, which infect *Pseudomonas aeruginosa*, actively inhibit their host's CRISPR-Cas systems. These phages produce anti-CRISPR (Acr) proteins that interact with components of the type I-F CRISPR-Cas interference machinery: e.g. the phage proteins AcrF1 and AcrF2 bind different subunits of Cascade and thus prevent the binding of the Csy complex to the target DNA. AcrF3 was shown to bind the nuclease Cas3, inhibiting its function in target degradation. Similar proteins were found to prevent type I-E CRISPR-Cas immunity in the same organism, thus raising the question whether Acr proteins exist for other CRISPR-Cas types [20,80–82].

In a so-far unique report of immune evasion, it has been shown that *Vibrio cholerae* ICP1 phages encode a type I-F CRISPR-Cas system that targets a host genomic island, known to be involved in CRISPR-unrelated anti-phage defence. Attacking the host's defence mechanism was crucial for phage propagation as the efficiency of infection was greatly reduced when targeting spacers in the viral CRISPR array were deleted. Intriguingly, analysis of phages that still managed to successfully infect the host acquired new spacers that originated from the same genomic locus, thus showing that the virus harbours a fully functional CRISPR-Cas system that is also active in acquisition [83].

## 4. Beyond adaptive immunity

Besides their role in prokaryotic immunity, CRISPR-Cas systems have been shown to participate in cellular pathways other than immunity.

### (a) DNA repair

Early reports suggested an involvement of the *E. coli* Cas1 protein in DNA repair pathways since the protein was shown to interact with components of the cellular repair machinery like RecB, RecC and RuvB. Cas1 further processed intermediate DNA structures that often occur during DNA repair and recombination like Holliday junctions, replication forks and 5′-flaps. Moreover, deletions of the *cas1* gene resulted in increased sensitivity towards DNA damage and affected chromosome segregation [14]. Moreover, the participation of the RecBCD recombination system in CRISPR-Cas immunity has become more evident in recent years. The RecBCD complex recognizes double-strand DNA (dsDNA) breaks that often occur at replication forks. After recognizing damaged DNA, RecBCD subsequently degrades the DNA until it reaches a Chi site [84]. A recent study suggested that the degradation products of the repair complex serve as templates for spacer acquisition as new spacers were mainly acquired from regions that lie in close proximity of stalled replication forks. With regard to antiviral immunity, RecBCD might be the first line of defence as it recognizes and degrades linear phage DNA and thus enables the adaptation machinery to collect new spacers. Acquisition from chromosomal DNA is prevented due to the frequent distribution of Chi sites within the host genome [85]. The requirement of RecB for type I-E CRISPR-Cas immunity was ultimately proven by another study demonstrating that a *recB* deletion abolished naive spacer acquisition in *E. coli*. Interestingly, the absence of RecB did not affect primed spacer acquisition. Here, the helicases RecG and PriA were essential, whereas DNA polymerase I was crucial for both, naive and primed adaptation [86]. The emerged model suggests that, during primed adaptation, RecG and PriA recognize the R-loop structure that occurs by binding of the Cascade : crRNA complex to the DNA. As a result, Cascade dissociates from the DNA leading to the exposure of temporary single-stranded DNA regions that may stimulate spacer acquisition by Cas1 and Cas2. As Cas3 is essential for primed adaptation, the nuclease activity of the protein is likely to promote the generation of DNA fragments that are captured by the acquisition machinery. During naive adaptation, DNA damage is possibly induced by Cas1 and leads to the recruitment of the RecBCD complex as described above [85,86].

### (b) Gene regulation

The involvement of CRISPR-Cas components in cellular regulatory processes became more evident in the last few years. Type II CRISPR-Cas systems seem to play a significant role in regulating virulence of pathogenic bacteria. In *Francisella novicida*, a ribonucleoprotein complex consisting of Cas9, tracrRNA and a unique small CRISPR-associated RNA represses the expression of a bacterial lipoprotein (BLP). Transcriptional downregulation of BLP is crucial for immune evasion as the protein can be recognized by the host's immune system. It is assumed that the ribonucleoprotein complex binds the *blp* transcript leading to degradation of the mRNA by Cas9 or an unknown nuclease. As a consequence, BLP is underrepresented on the cell surface resulting in a reduced immune response [87]. Similarly, *Neisseria meningitidis* mutant strains that lack a *cas9* gene showed severe survival defects in human epithelial cells [87]. In *Campylobacter jejuni*, *cas9* deletion mutants displayed less cytotoxicity in human cell lines. Presumably, the absence of *C. jejuni* Cas9 affects the biochemical composition of the bacterial cell wall and thus makes the cell more prone to antibody binding [88]. A type II-B Cas2 protein of *Legionella pneumophila* was crucial for infection of amoebae and thus represents another virulence-related function [89]. Transcription of an abandoned CRISPR array (no *cas* operon) in *Listeria monocytogenes* leads to the stabilization of a partially matching mRNA. Interestingly, if the CRISPR array is removed from the genome, the bacteria were able to colonize a murine liver more efficiently providing evidence for a regulatory function in virulence by an antisense RNA mechanism [90].

Endogenous regulation by CRISPR-Cas can also affect group behaviour in a bacterial population as identified in the life cycle of *Myxococcus xanthus*. The δ-preoteobacterium is able to produce myxospores to overcome environmental stresses like nutrient deficiency. Myxospores are produced during a complicated process that involves cooperated movement and aggregation of cells within a population. As a result, cell aggregates differentiate into the so-called fruiting bodies that contain the spores. *Myxococcus xanthus* possesses a type I-C CRISPR-Cas system and deletions of *cas7* and *cas5* lead to highly decreased sporulation. The same was true for a *cas8c* deletion that additionally resulted in delayed cell aggregation. Moreover, Cas8c stimulates synthesis of FruA, an important protein in the sporulation pathway [91–93]. The mechanistical involvement of Cas proteins in the formation of the fruiting body remains puzzling as a recent study added yet another level of complexity by demonstrating the involvement of a type III-B CRISPR array in fruiting body development and production of exopolysaccharides [94].

### (c) Genome evolution

The acquisition of foreign DNA spacers is a crucial step in CRISPR-Cas immunity and displays the unique adaptive nature of this defence system. It has been widely reported that in some cases, spacers are derived from own genomic sequences. Targeting of the chromosome, however, results in DNA damage and will inevitably kill the bacterial cell. While self-targeting of CRISPR-Cas systems can definitely be lethal for a host organism, several studies investigated its potential role in genome evolution. Besides small-scale genetic modifications like mutations in chromosomal PAM sequences, protospacers or the *cas* operon, large genomic rearrangements were observed in *Pectobacterium atrosepticum* when spacers matched sequences in the own genome. Here, a genomic island of approximately 100 kb that is involved in plant pathogenicity was remodelled or deleted [95]. A genomic study on *Thermotogales* revealed the association of CRISPR loci to sites of DNA inversions and other genetic rearrangements. Even though the exact involvement of the CRISPR arrays in fostering the observed genetic alterations remains unknown, CRISPR seems to promote these evolutionary events [96]. By contrast, one bioinformatic study suggested that self-targeting of CRISPR-Cas systems is a rather undesirable effect, because it conveys autoimmunity. As an outcome, CRISPR-Cas systems become degenerated due to mutations in *cas* genes and the target sites or by the inactivation or deletion of whole CRISPR-Cas systems which, indeed, promotes evolutionary variations but simultaneously leads to a loss of fitness regarding antiviral defence [97].

## 5. Significance and applications

The use of CRISPR-Cas in therapeutic approaches has become increasingly relevant in different fields of medicine. The presence of repetitive sequences interspersed with short spacers, later known as CRISPR, has been exploited for diagnostic purposes and simple typing of *Mycobacterium tuberculosis* strains [98]. This helped to understand ways used by pathogens for their transmission by looking at differences in the spacer content of related strains [98,99]. This so-called spoligotyping (spacer oligotyping) has also been adapted for *Salmonella enterica*, *Yersinia pestis* and *Corynebacterium diphteriae* [100–105].

The use of CRISPR-Cas as a direct antimicrobial tool has been studied recently. Artificial CRISPR arrays have been designed to kill pathogenic bacteria by targeting antibiotic resistance or virulence genes. This elegant way only aims for harmful strains in a bacterial population and allows non-pathogenic strains to overgrow the pathogens [106–108]. A recent study used lysogenic phages to introduce a CRISPR-Cas system in *E. coli*, which targets antibiotic resistance genes. The array was designed to additionally target the genomes of lytic phages leading to immunity towards phages only of antibiotic-sensitized bacteria. More precisely, bacteria that are unlikely to acquire antibiotic resistance genes due to their engineered spacer content are also resistant to lytic phages. Thus, in the case of a phage infection, only pathogenic strains would be eradicated from the population [109].

The medical potential of the CRISPR-Cas systems goes beyond antimicrobial treatment. The introduction of efficient and precise modifications into genes of an organism displays the basis for genome engineering. Programmable nucleases are used that specifically bind genomic regions and cleave the DNA at a desired position. Zinc finger nucleases (ZFN) and transcription activator-like effector nucleases (TALENs) have been widely used to edit DNA. Both genome editing tools rely on the same principle: a sequence-specific DNA binding domain, which provides specificity, is fused to a nuclease. Because of its simplicity, effectiveness and the possibility to target multiple genomic sites simultaneously, use of the CRISPR-Cas9 system is usually favoured over ZFN and TALEN systems [110–112]. The bacterial defence protein Cas9 is used to target almost any desired DNA sequence with the help of a targeting RNA. This single-guide RNA (sgRNA) is an engineered hybrid of the naturally occurring tracrRNA:crRNA duplex and thus simplifies its application for genome editing purposes [45].

**Figure 2.**
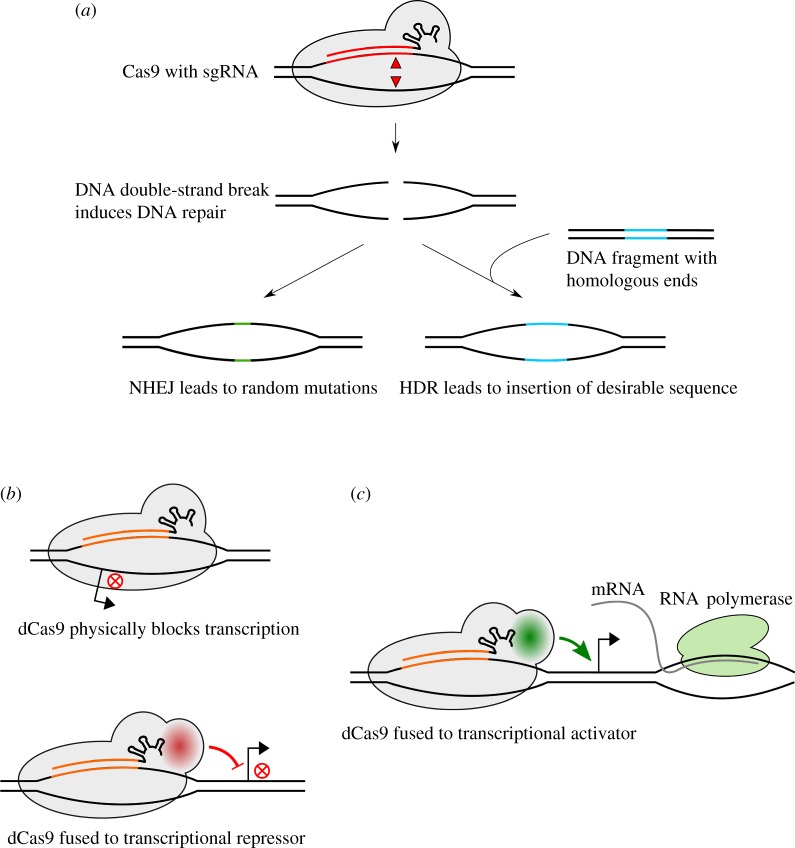
Applications of the CRISPR-Cas9 technology. (*a*) Cas9 is guided by a sgRNA to induce a double-strand DNA break at a desired genomic locus. The DNA damage can be repaired by NHEJ yielding short random insertions or deletions at the target site. Alternatively, a DNA sequence that shows partial complementarity to the target site can be inserted during HDR for precise genome editing purposes. (*b*) Mutations in the catalytical domains of Cas9 yield a dead variant (dCas9) that binds but does not cleave DNA. The approach with dCas9 is used for transcriptional repression by binding to the promoter region of a gene and thus blocking the access for the RNA polymerase. Similarly, dCas9 can be fused to a transcriptional repressor. Red crosses represent inhibition of transcription. (*c*) The fusion of dCas9 to a transcriptional activator stimulates transcription of an adjacent gene by recruiting the RNA polymerase.

Repurposing the CRISPR-Cas9 system for genome editing exploits the DNA repair mechanisms of eukaryotic cells: after the introduction of a double-strand DNA break, the cell can repair the damage by non-homologous end joining (NHEJ). This process is error-prone and often leads to point mutations, deletions or causes frameshifts that alter the gene product and eventually abolishes its function, which is favoured for genetic knockouts. Precise genome engineering, however, relies on another pathway, termed homology-directed repair (HDR), where a piece of DNA that shows sequence homology to the target site is used to repair the DNA via homologous recombination. This short DNA sequence can harbour any sort of insertion or alteration, allowing the integration of any desirable DNA sequence at the target site [50,113–117] (figure 2*a*).

Mutations in the nuclease motifs of Cas9 lead to a ‘dead’ variant that is unable to cleave DNA and thus can be used to regulate transcription of a desired gene. By targeting the promoter region or the open reading frame of a gene, binding of the RNA polymerase is physically blocked and mRNA elongation is inhibited. Alternatively, dCas9 can be fused to a repressor that controls gene transcription (figure 2*b*). Transcriptional activation can be achieved by fusing dCas9 to a transcription activator that recruits the RNA polymerase and induces gene expression (figure 2*c*). In some cases, gene knock-downs are desired over gene knockouts, e.g. if the targeted gene is essential [116,118–122]. The CRISPR-Cas9 method has also been exploited for epigenome editing that allows the control of gene expression by introducing modifications like DNA methylation or histone acetylation. One study showed that a fusion protein of dCas9 and the core domain of the human acetyltransferase p300 could activate gene expression at specific sites [123]. Moreover, fusion of dCas9 to the KRAB repressor was able to induce methylation at specific enhancers leading to reduced chromatin accessibility and, thus, silencing of gene expression [124]. Precise epigenome editing has great potential to reveal site-specific chromatin modification and helps to explore the regulation of gene expression that could lead to new therapeutical strategies. Other approaches—mainly in prokaryotes—exploit the endogenous type I effector complex Cascade for similar experiments if the nuclease Cas3 is absent [125–127]. In all of the aforementioned genome manipulation strategies, the existence of a PAM adjacent to the target site is a strict requirement [9].

Precise remodelling of the genome can be used to cure gene variants that cause genetic diseases. Scientists were able to repair mutations that cause cystic fibrosis (CF) by correcting the *cftr* locus in cultured intestinal stem cells of CF patients [128]. Moreover, using the CRISPR-Cas9 technique, a healthy phenotype could be restored in mice suffering from hereditary tyrosinaemia, a genetic disease that causes severe liver damage [129]. Genome editing has been further used to develop antiviral therapeutic approaches. Accordingly, the genome of HIV has been successfully eradicated from latently infected cells [130]. Indeed, a recent study demonstrated that the generation of NHEJ-induced mutations in the viral genome led to replication defects of the virus. However, it also drove the generation of replication competent mutants that harbour mutations at the target site and thus are no longer targeted by Cas9 [131]. Other studies aim to alter a specific surface protein called CCR5 that serves as a co-receptor for the HI-virus to enter a host cell. Mutations in the *ccr5* gene can prevent the virus from infecting a cell leading to a highly resistant but otherwise healthy phenotype. In fact, altering the wild-type *ccr5* gene leads to immunity of monocytes and macrophages against HIV infections [132–134].

The CRISPR-Cas9 technique has further simplified genome-scale screens. These screens seek to identify genes that are involved in certain metabolic or pathogenetic processes by abolishing gene function and studying the resulting phenotype. Using this approach, genes that are involved in tumour growth [135] or convey susceptibility towards bacterial toxins [136] could be identified. Previously, RNA interference (RNAi) was used to knock down gene expression in a sequence-specific manner. However, RNAi only decreases the abundance of transcripts, whereas CRISPR-Cas9 enables a full knock-out of candidate genes. Moreover, multiplexing (targeting of several genetic loci at the same time) is crucial for this approach and can be achieved by using a library of different sgRNAs that is usually delivered with Cas9 by a lentiviral vector system [135–138].

## 6. Perspectives

Interest in the field of CRISPR-Cas has rapidly increased in recent years. Numerous studies shed light onto the underlying genetic and biochemical processes of the adaptive prokaryotic immune system thus revealing its potential in modern medicine (box 2). Undoubtedly, the versatility of different CRISPR-Cas systems is stunning and with the recent discovery of three new types we may have just begun to fully understand the significance of CRISPR-Cas as a microbial defence system. However, many aspects of the antiviral system require further insight. The process of immunization that is accomplished by incorporation of new spacers in the CRISPR array is still the most puzzling event in CRISPR-Cas immunity. The precise biochemical basis of spacer acquisition and its degree of conservation among the different types has yet to be uncovered. For instance, the function of additional proteins like Cas4 and Csn2 that have been shown to be required for adaptation needs further investigation. Primed adaptation has only been observed in type I CRISPR-Cas systems even though this process provides a great protective advantage towards mutated phages that would escape CRISPR interference.

Box 2. **Milestones in CRISPR-Cas research.**

First described in 1987 as unusual repetitive sequences [139], the interest in CRISPRs and their associated genes slowly increased throughout the 1990s and early 2000s. Initially believed to participate in cellular DNA repair and replicon partitioning processes, first evidence that CRISPR-Cas systems display an adaptive prokaryotic immune system was delivered in 2005 [4]. Researchers were surprised as they found that most of the interspersed sequences interspaced between identical repeats derived from extra chromosomal DNA, more specifically from phage genomes and conjugative plasmids [4,100,140]. The hypothesis was eventually proven two years later when scientists showed the incorporation of new spacers into a CRISPR-Cas locus of *Streptococcus thermophilus* after challenging the bacterium with a bacteriophage. The newly acquired spacers always showed perfect complementarity to sequences on the phage genome and conveyed resistance towards that particular phage upon a subsequent infection [1]. Research interest of the CRISPR field soon accelerated, leading to new discoveries that helped to understand the basic mechanisms of the immune system. In 2008, the processing of the CRISPR transcript into mature crRNAs that guide the Cascade complex of the *E. coli* type I-E system was experimentally validated, also giving hints that DNA rather than RNA is targeted [54]. The latter was confirmed in the same year as a study demonstrated that indeed DNA is the targeted molecule [56]. This led scientists to think about the potential role that this prokaryotic immune system might play as a DNA manipulation tool. Today, CRISPR-Cas9 is a frequently harnessed tool for genome editing purposes and major progress in understanding the underlying biochemical processes in RNA-guided Cas9 was presented in recent years. In 2010, researchers showed that Cas9 creates a single double-stranded break at a precise position on the target DNA [63]. Further insight into the mechanism was delivered 1 year later as the involvement of another small RNA, called tracrRNA, was shown. The maturation of crRNA requires tracrRNA as well as Cas9 and RNase III [42]. Evidence that the system would function heterologously in other bacteria was demonstrated in 2011, as the *S. thermophilus* type II CRISPR-Cas system could provide immunity in *E. coli* [141]. Other research had shown certain elements of the type II system, including the involvement of a PAM sequence in interference [6,26,141] but the nature of the cleavage complex remained unknown. In 2012, tracrRNA, which was previously known to be involved in crRNA maturation [42], was shown to also form an essential part of the DNA cleavage complex, with the dual tracrRNA:crRNA directing Cas9 to introduce double-strand breaks in the target DNA [45]. Further simplification of the programmed targeting was achieved by creating a single-guide RNA fusion of tracrRNA and crRNA, that guides Cas9 for sequence-specific DNA cleavage [45]. A few months following the description of the CRISPR-Cas9 technology [45], a number of publications demonstrated its power to edit genomes in eukaryotic cells and organisms, including human and mouse cells [116,117].

Another puzzling aspect is the impact of CRISPR-Cas systems on prokaryotic diversity. It has been observed that the immune systems protect not only against phages, but also against other MGEs that might have beneficial effects for an organism. In fact, the native CRISPR-Cas system is silenced in *E. coli* by the histone-like nucleoid structuring protein H-NS [142], raising the idea that an inactive system may be advantageous for the bacteria. In addition, CRISPR-Cas systems can interfere with plasmid conjugation and transformation of naturally competent bacteria [43,143]. Several studies show a negative correlation between the occurrence of CRISPR-Cas systems and the amount of MGEs within the chromosome, which seems like a limitation to evolutionary processes and horizontal gene transfer (HGT) [144,145]. Contradictory results were presented by an evolutionary analysis that found no significant correlation between the activity of a CRISPR-Cas system and the number of HGT events [146]. However, these relations have to be assessed in context with further factors like predatory pressure, the occurrence of other defence systems and the fitness costs that are connected to the maintenance of adaptive defence. It has been stated that bacteria may lose or inactivate their CRISPR-Cas systems when they face a high abundance of predators. In such environments, phage resistance due to, for example, receptor mutations seems to be more affordable [147]. More precisely, high viral mutation rates render adaptive immunity obsolete as the costs of adapting to a dynamic predatory habitat exceed the immunological benefits [148]. Interestingly, another study showed that simply maintaining a CRISPR-Cas system without any predatory pressure can result in an adverse balance regarding fitness costs. Here, a wild-type strain showed reduced competitive capabilities compared with a *cas* gene knockout mutant. In the case of phage infection, however, no increased fitness costs were observed as described above [149] and, thus demonstrating that these dynamic phage–host interactions are highly complex and need more elaboration in future scientific work.

Further research is also required on immunity-unrelated functions of CRISPR-Cas systems. Numerous studies revealed their involvement in several regulatory processes (see §4) and deeper insight is needed, for instance, when it comes to the interaction of Cas proteins with components of cellular DNA repair and recombination pathways.

Besides their fascinating role in prokaryotes, CRISPR-Cas systems undoubtedly caught most attention for their potential in medical applications and numerous other biotechnological applications like crop editing, gene drives (the ability to stimulate biased inheritance of particular genes to alter an entire population) and synthetic biology (non-medical applications are not discussed here; see [150] for details). Despite the enormous potential that lies within the CRISPR-Cas9 technology, further investigation is required to make the system an applicable and safe tool for therapeutically useful approaches. Challenging issues that remain and need to be addressed in the future include off-target cleavage by Cas9. Off-target effects are a major concern when precisely remodelling the genomic content of eukaryotic cells. In some cases, genetic alterations at off-target sites were detected at higher frequencies than the desired mutation which clearly reveals the need for higher specificity of the technique [151]. Strategies preventing off-target effects include the injection of purified Cas9 directly into a cell instead of expressing the recombinant protein in the target cell. This method is convenient for fast target cleavage but also leads to the rapid decay of Cas9, thereby reducing the possibility of off-target effects [152,153]. Moreover, using two sgRNAs that target both strands of the target sequence in combination with a DNA-nicking variant of Cas9 was shown to reduce off-target effects significantly [154]. Further strategies focus on optimizing sgRNA sequences in order to achieve more reliable editing. Truncating sgRNAs by 2–3 nt was shown to improved target specificity [155]. Also, adding two guanine nucleotides at the 5′ end, directly next to the target-complementary region of the guide RNA, could reduce off-target effects [156]. Another issue that requires further investigation is the overall delivery of the CRISPR-Cas9 system into desired cells of a multicellular organism. Promising *in vivo* approaches include viral and non-viral vector systems that deliver Cas9 and sgRNA to the desired cells [110,157]. Moreover, *ex vivo* concepts rely on isolating patient-derived cells which are transplanted back after genomic editing. A major advantage in using this approach is the assessment of the genetic alteration that was introduced. Here, only correctly edited cells without malign off-target mutations are chosen for transplantation [110,157]. Although some challenges remain, it only seems to be a matter of time until CRISPR-Cas9-based genome editing will become a safe and applicable method used in a variety of therapeutic approaches.
